# Characterization of Cyclic Olefin Copolymers for Insulin Reservoir in an Artificial Pancreas

**DOI:** 10.3390/jfb14030145

**Published:** 2023-03-04

**Authors:** Norma Mallegni, Mario Milazzo, Caterina Cristallini, Niccoletta Barbani, Giulia Fredi, Andrea Dorigato, Patrizia Cinelli, Serena Danti

**Affiliations:** 1 Department of Civil and Industrial Engineering, University of Pisa, Largo Lucio Lazzarino, 56126 Pisa, Italy; 2 National Interuniversity Consortium for Materials Science and Technology (INSTM), Via Giuseppe Giusti 9, 50121 Florence, Italy; 3 Institute for Chemical and Physical Processes (IPCF), National Council of Researches (CNR), Via Giuseppe Moruzzi 1, 56126 Pisa, Italy; 4 Department of Industrial Engineering, University of Trento, Via Sommarive 9, 38123 Trento, Italy

**Keywords:** diabetes, thermal characterization, mechanical characterization, additive manufacturing, 3D-printing, Topas, surface roughness, insulin aggregation

## Abstract

Type-1 diabetes is one of the most prevalent metabolic disorders worldwide. It results in a significant lack of insulin production by the pancreas and the ensuing hyperglycemia, which needs to be regulated through a tailored administration of insulin throughout the day. Recent studies have shown great advancements in developing an implantable artificial pancreas. However, some improvements are still required, including the optimal biomaterials and technologies to produce the implantable insulin reservoir. Here, we discuss the employment of two types of cyclic olefin copolymers (Topas 5013L-10 and Topas 8007S-04) for an insulin reservoir fabrication. After a preliminary thermomechanical analysis, Topas 8007S-04 was selected as the best material to fabricate a 3D-printed insulin reservoir due to its higher strength and lower glass transition temperature (T_g_). Fiber deposition modeling was used to manufacture a reservoir-like structure, which was employed to assess the ability of the material to prevent insulin aggregation. Although the surface texture presents a localized roughness, the ultraviolet analysis did not detect any significant insulin aggregation over a timeframe of 14 days. These interesting results make Topas 8007S-04 cyclic olefin copolymer a potential candidate biomaterial for fabricating structural components in an implantable artificial pancreas.

## 1. Introduction

Type-1 diabetes mellitus (T1DM) is one of the most common metabolic disorders. It consists of a dysfunction of the pancreatic β-cells, which induce a significant deficiency of insulin and consequent hyperglycemia [1]. The traditional approach to treat T1DM is the recurrent injection of insulin throughout the day, a strategy that has been considered suboptimal since it cannot provide a finely tuned glucose profile, as in healthy conditions. In fact, it leads to an uncontrolled oscillation of hypo-/hyperglycemic occurrences, a scenario that has been demonstrated to be critical in the long term [2].

Several approaches have been proposed to obtain an optimal glucose profile in diabetic patients, including surgical approaches (i.e., pancreas or islet transplantation) that, however, are limited by the shortage of organ donors and the need for an immunosuppressive drug therapy to avoid rejections [3,4]. A different avenue consists of the development of an artificial organ [5], namely, an artificial pancreas (AP). This appears definitely to be an intriguing approach, since it would allow a closed-loop control of glucose and the reduction in extra needs given by peaks/shortage of insulin [6,7]. In recent years, APs have been classified based on the body placement (i.e., external, wearable vs. internal, implantable), or on the insulin route (i.e., subcutaneous vs. intravenous vs. intraperitoneal, the most reliable for the expected outcome) [8,9]. Independently of the architecture, the final goal of an AP is to recover a normal lifestyle in patients with a low-impact solution. In view of this challenging goal, researchers have been developing autonomous and implantable APs, facing several issues, including, but not limited to, anatomical and biological constraints, insulin storage and aggregation, efficient pumping, and algorithmics modeling for glucose monitoring in closed loop [10,11,12,13]. The reservoir of insulin, however, has been considered one of the most critical components for an efficient AP, since it must safely store insulin, thus preventing insulin degradation and aggregation, which would render the entire system unsuitable because of clot formation. Insulin is, indeed, a delicate biomolecule, which can aggregate due to multiple factors, including temperature, mechanical stress, and contact with air [14,15,16]. Among the noted critical conditions, some of them cannot actually be controlled, as they depend on aleatory in-body conditions (i.e., mechanical agitation, temperature). Therefore, although researchers have developed stable insulin formulations [17], a new research avenue consists of the search for a constitutive material for the reservoir, inherently able to prevent the formation of insulin clots [18,19].

Specifically, some main features are important to be characterized, namely, the material chemistry [20,21] the surface texture and the mechanical stirring, since they primarily affect the insulin-material behavior [22,23], including those in which they are used as thermoplastics to heal epoxy resins. Iacovacci et al. have recently investigated the possibility to fabricate this component with Nylon 6 and Polytetrafluoroethylene (PTFE), showing, through a mechanical characterization and UV spectra analysis, that a smooth Nylon 6 coating is a good insulin reservoir candidate, as it favors insulin stability by preventing localized aggregations [13].

**Figure 1:**
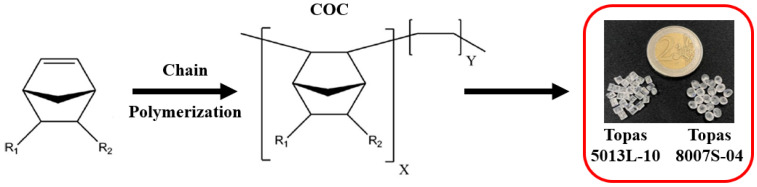
Schematic of the synthesis of Topas 5013L-10 and Topas 8007S-04 COCs through chain polymerization; X = norbornene, and Y = olefin units; R = functional group.

In this work, we present a different approach for fabricating insulin reservoirs, using cyclic olefin copolymers (COCs). COCs are a family of materials obtained through the chain copolymerization of cyclic monomers, such as commercial Topas^®^ or Apel^®^). This process is an alternative to the ring-opening metathesis polymerization of cyclic monomers followed by hydrogenation that leads to cyclic olefin polymers (COPs) such as Zeonex ^®^ or Zeonor^®^. In both cases, different materials can be achieved depending on the cyclic monomer and the polymerization process used for their synthesis [24]. COCs show remarkable physico-chemical properties, such as glass-like transparency, rigidity, heat and chemical resistance, and low permeability to gas and water. These features make COCs potential candidates for a large number of applications [24,25,26,27,28,29], including those in which they are used as thermoplastics to heal epoxy resins [30,31,32]. In the biomedical field, COCs have been employed as materials for drug packaging (i.e., blisters), as liquid blends for injectable formulations [27,33,34,35], and as polyethylene coatings for bone replacement applications [36]. In our study, we approach the design of an insulin reservoir by investigating the suitability of two COC grades, namely, the Topas 5013L-10 and Topas 8007S-04, as constitutive material candidates. Before addressing the suitability of the material for insulin storage, we characterized both COCs from a thermomechanical standpoint to select the best candidate for the subsequent additive manufacturing process (Figure 1). 

## 2. Materials and Methods

### 2.1. Materials

Two COCs were used: Topas 5013L-10 (T5013) and Topas 8007S-04 (T8007), based on their potential employment in a biomedical prosthetic scenario. Both materials are purchased from Advanced Polymers (Frankfurt, Germany and Florence, CA, USA) in the form of granules. As reported in Figure 1, the T5013 granule had a cylindrical shape with dimensions of ϕ 2.09 ± 0.11 mm × 2.94 ± 0.22 mm whereas the T8007 granule has an odd round shape with dimensions of 3.13 ± 0.19 mm. The two commercial grades of COCs used in this study contain a norbornene content of 65 w% and 75 w% for the T8007 and T5013, respectively [37]. Insulin injectable solution for human use (Humalog^®^ 100 U/mL) was supplied by Eli Lilly Itala S.p.a. (Sesto Fiorentino, FI, Italy).

### 2.2. Mechanical Characterization

The two COCs were extruded in a co-rotating conical twin-screw extruder Minilab II HaakeTM Rheomex CTW 5 (Thermo Fisher Scientific, Waltham, MA, USA). The molten material was then transferred to a Thermo Scientific Haake Minijet II (Thermo Fisher Scientific, Waltham, MA, USA). Tensile tests were performed using dog-bone tensile bars molded through injection using the Haake Type 3 (557-2290), following the protocols reported in ASTM D 638. The fabrication conditions used for both samples are reported in Table 1. 

Tensile tests were carried out using the Instron 5500R universal testing machine (Canton, MA, USA) equipped with a load cell of 10 kN and a crosshead speed of 10 mm/min. Data were collected with the TestWorks 4.0 software (MTS Systems Corporation, Eden Prairie, MN, USA).

Following the standard method ISO 179:1993, Charpy’s Impact test samples (80 × 10 × 4 mm^3^ parallelepiped) are performed on V-notched specimens using a 15 J Charpy pendulum (CEAST 9050, Instron, Canton, MA, USA).

For each mechanical test, five replicates (*n* = 5) were tested at room temperature (RT) for each sample.

### 2.3. Thermal Characterization

A first assessment of the thermal properties was performed using thermogravimetric analysis (TGA) and differential scanning calorimetry (DSC). TGA measurements were carried out in duplicate (*n* = 2) on 20 mg of sample, using the Q500 TGA (TA Instruments, New Castle, DE, USA), under nitrogen flow (60 mL/min) and a heating speed of 10 °C/min from RT to 600 °C. The thermogravimetric (TG) and derivative thermogravimetric (DTG) curves for the two COC grades were performed at a constant heating rate of 10 °C/min. The onset temperatures (T_os_) of degradation were obtained from the TGA curves by extrapolating back to the initial weight of the polymer from the curve at the peak of degradation. We estimated the end temperature of degradation (T_e_) from the TGA curve by extrapolating the degradation peak. This parameter and the associated temperature (T_p_) were calculated from the DTG plot thermograms at the maximum rate of weight loss.

DSC measurements were performed with a DSC Q200 (TA Instruments, Waters LLC, New Castle, DE, USA) calibrated with standard indium. Both the samples (12 mg) were first heated from RT to 300 °C at a heating rate of 10 °C/min to cancel any previous thermal history. After an isotherm profile at 300 °C for 2 min, the samples were cooled down and kept at −70 °C for 5 min, before being heated again up to 300 °C with an increasing temperature rate of 10 °C/min.

A second assessment concerned the evaluation of the thermal diffusivity (α), specific heat capacity, and conductivity that were measured at 25 °C with a light flash apparatus LFA 467 Hyperflash (NETZSCH-Gerätebau GmbH, Selb, Germany). Round samples with a diameter of 12.7 mm were die-cut from compression-molded 2 mm thick sheets and sprayed with graphite. At least two specimens per sample (*n* = 2) were tested, and five pulses are performed on each specimen. The thermal diffusivity (α) was determined using the Transparent method with a pulse correction (software Proteus V.8.0.2); the specific heat capacity (C_P_) was determined using Pyroceram 9606 as the reference material. Finally, the thermal conductivity (λ) was calculated as the product of α, C_P_, and the gravimetric density, equal to 1.02 g/cm^3^ for the T5013 and 1.01 g/cm^3^ for the T8007, respectively, as reported on the technical datasheets.

### 2.4. Design and Fabrication of Reservoir

A reservoir was designed aimed at providing a structure to test the quality of the surface finishing (aka roughness) and fabricated with an additive manufacturing approach. The produced samples were also used to assess possible insulin aggregation. 

**Figure 2:**
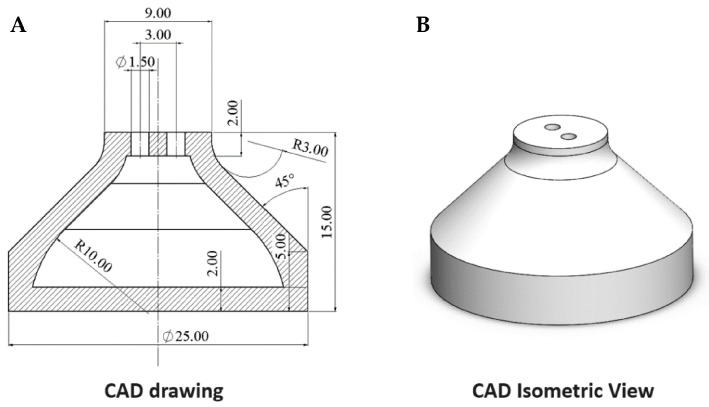
(**A**) 2D CAD drawing, and (**B**) isometric view of the Topas-based reservoir.

Figure 2A reports the design of the reservoir in the form of a 2D CAD drawing and Figure 2B reports a trimetric 3D view. In particular, the reservoir was designed with a cone-shaped structure (slope of about 45°) to minimize the material content and, in parallel, to ensure a good printed outcome. The structure that will be filled with diluted insulin possesses two holes on the top surface to enable an efficient flushing of the tank with nitrogen, needed to prevent air from being in contact with insulin. 

Topas filaments were made using a 3devo maker system (Utrecht, The Netherlands), heating the extruder up to 195 °C and using a processing speed of 3.5 rotations per min (rpm). The fabrication of the reservoir was performed with a filament-based Ender3 Pro (Creality, Shenzhen, China), equipped with a dyze extruder (LeMoyne, Quebec City, QC, Canada). The filament and the printing bed were heated up to 265 °C and 80 °C, respectively, while the deposition velocity was set up to 50 mm/s.

A geometrical assessment was performed using a caliper to evaluate potential deviations on the main topological features, namely the bottom diameter, the height, and the diameter of the top-surface holes. Surface finishing was measured using a Mitutoyo SJ-310 (Mitutoyo Italiana S.r.l., Lainate, Milano, Italy) on three locations of the structure positioned on the bottom and lateral surfaces. We acquired profile lengths of about 1 mm and estimated the roughness in terms of R_a_, defined as the arithmetic average of profile height deviations from the mean line.

### 2.5. Insulin Aggregation Tests

To evaluate the ability of the material to prevent insulin aggregation, the ultraviolet (UV) spectra of insulin solutions from samples collected over 14 days were assayed. Specifically, the protocol consisted in pouring 4 mL of insulin (Humalog 100 U/mL equals to 3.5 mg/mL) into the fabricated tank, flushed with gaseous nitrogen at 70 mL/min. Therefore, the tank was firmly sealed and kept at 35 °C. At each observation point (day 0, 1, 7, 10, and 14), a total of 100 µL of insulin was collected and diluted to 0.1 mg/mL for the UV-spectrophotometric analysis. The absorbance (A) values were observed at two different wavelengths, 276 nm (A276) and 350 nm (A350). In particular, A276 allowed the transparency of the solution to be observed, thus enabling the evaluation of the insulin concentration. In contrast, A350 was associated with the formation of fibrils in insulin, thus, a null value of the absorbance corresponded to the absence of any aggregation [6]. As a reference, all results were compared to those of commercial insulin stored in their original glass sterile case. Additionally, we have performed a 7-day long experiment to assess the potential agglomeration of the insulin when the reservoir is mechanically agitated. We have placed the reservoir in a thermostatic bath at 37 °C endowed with and oscillating plate, and measured the absorbance at 350 nm at days 3 and 7.

**Figure 3:**
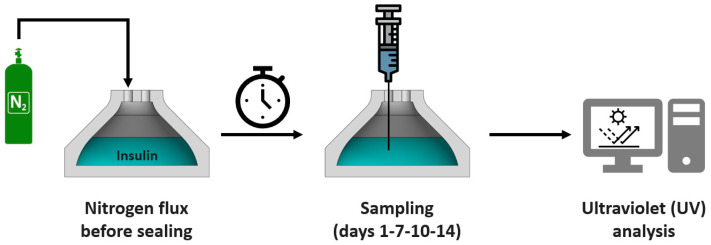
Schematic showing insulin storage set-up and aggregation tests. After filling the tank with insulin, a gaseous nitrogen was fluxed to remove the air present inside, before a firm sealing. On day 1, 7, 10 and 14, a sample of insulin was taken from the tank for UV assays. Between sampling days, the tank was kept firmly sealed with an inert atmosphere made of nitrogen.

Figure 3 reports schematically the protocol followed to assess the ability of the COC-made container to prevent insulin aggregation.

### 2.6. Statistical Analysis

Statistical analysis was carried out to discuss the significance of the differences observed between the T5013 and T8007, and between the groups of data collected for the insulin aggregation tests. Data were expressed as mean and standard deviation. Independent student *t*-test analyses were performed using the Jamovi Software (version 2.2.5), considering the numerosity of the samples, and setting a significance threshold of 0.05.

## 3. Results

### 3.1. Mechanical Characterization

**Figure 4:**
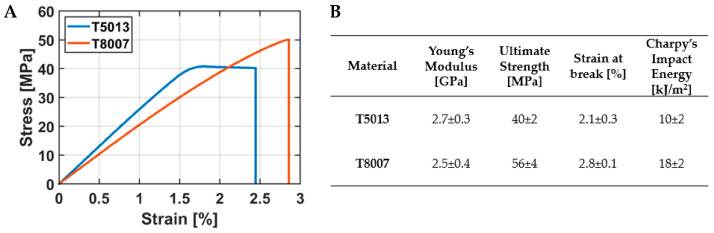
Mechanical characterization for the two COCs: (**A**) Graph showing the stress–strain curves for T5013 and T8007, and (**B**) table summarizing the obtained values for the properties measured with tensile and impact tests. Data are reported as mean ± standard deviation.

The mechanical characterization of the two COCs encompassed tensile tests and Charpy’s impact test, which enabled the evaluation of the Young’s modulus and the ultimate strength and strain, as well as of the energy stored before break, respectively. As reported in Figure 4, T5013 presented an elastic–fully plastic behavior with a higher stiffness than that of the T8007 (2.7 ± 0.3 GPa vs. 2.5 ± 0.4 GPa). Although the T8007 showed a lower elasticity, it possessed a higher yield stress than T5013, which corresponded to the ultimate stress of the material (56 ± 4 MPa vs. 40 ± 2 MPa). The strain at break for the T8007 was slightly higher than that of the T5013 (2.8% ± 0.1% vs. 2.1% ± 0.3%). These results, together with the estimated ultimate strengths matched the outcomes of the Charpy’s impact tests, in which a higher energy at break was observed for the T8007, with respect to that for T5013 (18 ± 2 kJ/m^2^ vs. 10 ± 2 kJ/m^2^).

### 3.2. Thermal Characterization

**Figure 5:**
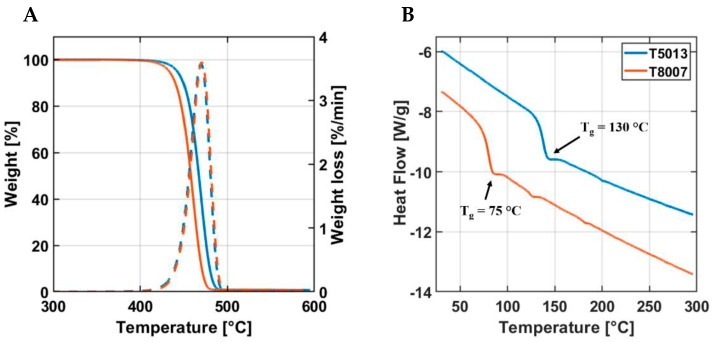
Thermal assessment of the two COC grades using TGA and DSC: (**A**) thermograms highlighting weight and weight loss, as a function of the temperature, and (**B**) heat flow as a function of the temperature with arrows indicating the glass transition temperature (T_g_).

The thermal assessment of the two COC grades encompassed the degradation temperature and thermal stability (assessed via TGA and DSC), as well as the conductivity, the specific heat, and the diffusivity. Figure 5 shows the main results obtained by TGA and DSC characterization. In particular, from the TGA analysis (Figure 5A), similar results for both the COC grades were detected, and specifically T_os_ ~ 450 °C, T_p_ ~ 470 °C, and T_e_ ~ 480 °C. From the DSC analysis, it was highlighted that T5013 had a higher heat flow across temperature than that of the T8007 (Figure 5B). Interestingly, the T_g_ values were lower for the T8007 (T_g_ = 75 °C) with respect to that of T5013 (T_g_ = 130 °C).

**Figure 6:**
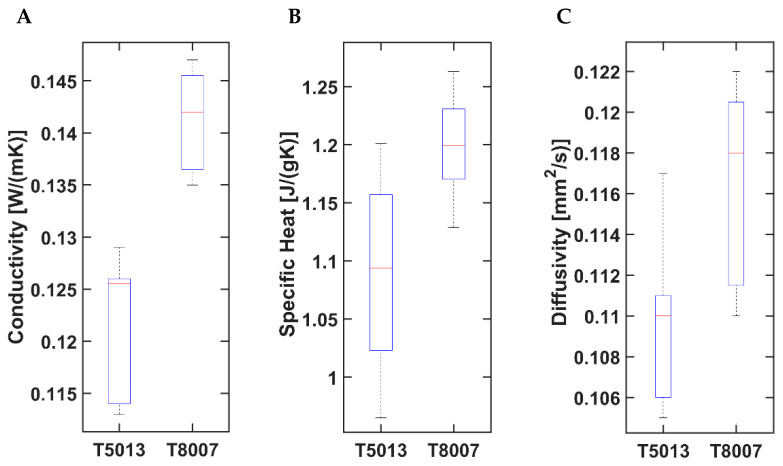
Bar graphs showing (**A**) thermal conductivity, (**B**) specific heat capacity, and (**C**) thermal diffusivity, for the two COC grades. The statistical analysis reports a significant difference between the materials for each thermal property with a *p*-value ≤ 0.001.

Figure 6 shows the outcomes of the other thermal properties. For all the considered parameters, namely, the conductivity, specific heat, and diffusivity, T8007 presented higher values, with statistical significance, than those of the T5013 (*p*-value ≤ 0.001).

### 3.3. Fabrication of the Insulin Reservoir

**Figure 7:**
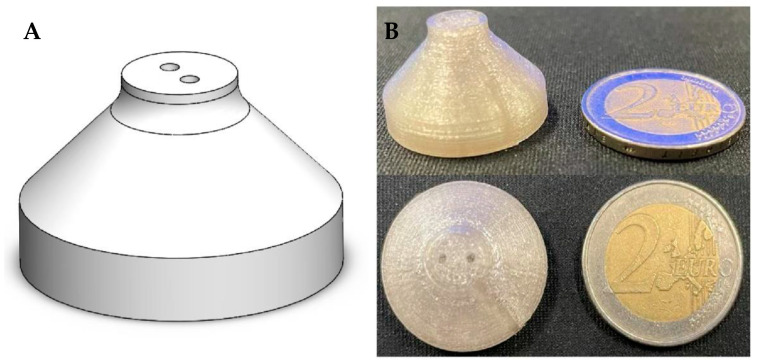
Fabrication of the reservoir to test the insulin anti-aggregation properties of the geometry/material: (**A**) CAD model, and (**B**) 3D-printed device (left); a 2 EUR coin for size comparison (right).

Based on the previous results, we fabricated the insulin reservoir with the T8007, since it possesses a lower T_g_ and higher mechanical properties than the T5013. Figure 7 shows the result of the fabrication process, comparing the CAD drawing and the actual structure obtained with the modeling approach.

**Figure 8:**
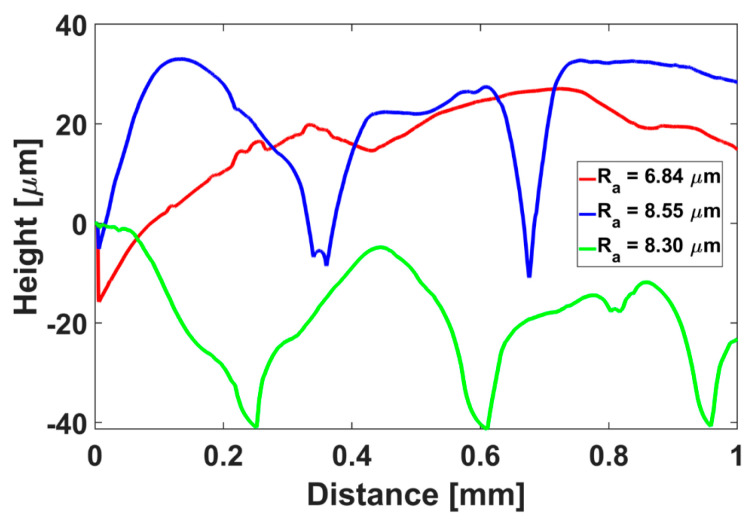
Roughness measurements on the surfaces of the insulin reservoir in three characteristic locations of the insulin reservoir.

Performing the geometrical assessment with the caliper on the main dimensions (i.e., height, base circumference, hole diameters on the top surface), an average dimensional error of 2% with a maximum deviation of about 3% for the top-hole diameters could be estimated. The assessment of the surface texture was analyzed using a digital optical profilometer. The analysis of three characteristic profiles displayed a roughness R_a_ in the range of 6.84–8.55 µm and localized variations between peaks and notches in a range of 0–40 µm, as reported in Figure 8.

### 3.4. Insulin Aggregation Tests

Insulin aggregation tests were performed using the UV spectrophotometric analysis at 276 nm (A276), corresponding to the insulin absorption peak, and at 350 nm (A350), corresponding to the region of total transparency of the substance. The results from the samples from the insulin reservoir were compared with those obtained from a reference liquid insulin stored in its sterile glass cartridge.

**Figure 9:**
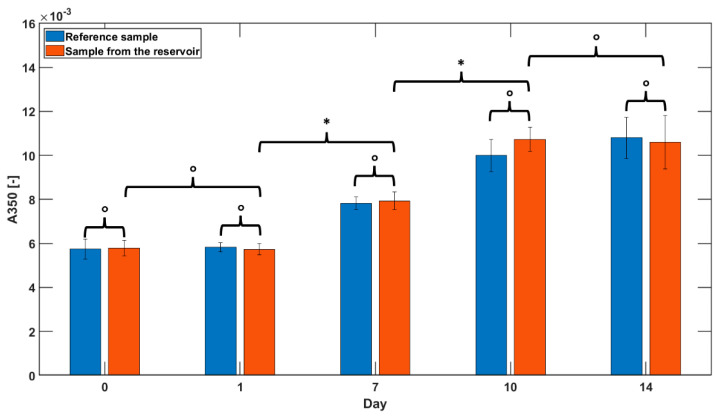
Bar graph reporting the outcomes of UV analysis performed at an absorbance of 350 nm (A350) to assess the aggregation of insulin at 0, 1, 7, 10, and 14 days comparing insulin samples stored in the fabricated T8007 reservoir (orange bars) and pristine insulin, used as reference (blue bars). Results from the statistical analysis are reported with the following symbols: ° *p*-value ≥ 0.317; * *p*-value ≤ 0.005.

While the A276 was almost insensitive to time (data not shown), the analysis of the A350 presented some relevant results. In Figure 9, a statistically relevant increasing trend in the A350 was observed for both samples after day 1 (*p*-value ≤ 0.005), which however gave values almost null (i.e., below 16 × 10^−3^). At each observation point, the comparison of the results between the sample and the reference did not report any significant statistical difference (*p*-values ≥ 0.317). 

As for the experiments conducted to evaluate the potential agglomeration of insulin under **a** mechanical agitation, we observed values of A350 of 10 × 10^−3^ ± 1 × 10^−3^ and 9 × 10^−3^ ± 2 × 10^−3^ at days 3 and 7, respectively, with no significant statistical difference (*p*-value ≤ 0.001). 

## 4. Discussion

An insulin reservoir is a main component of an implantable AP, the latter currently considered to be the way forward for future treatment of Type-1 diabetes [2]. This artificial organ is in fact expected to provide a fine glycemic control in the blood stream, keeping its values in line with a healthy condition throughout the day [7]. The insulin reservoir may a critical component for an AP, as it has to comply with three key requirements: (i) be made of a non-biodegradable biocompatible material able to be hosted by the surrounding tissues; (ii) safely store insulin, thus preventing its aggregation, i.e., via suitable chemistry and surface texture; (iii) be shaped with a macro-scale geometry that fits the stringent requirements for an efficient, safe and life-long implantation. The first issue has been explored by Cristallini et al. [38], who proposed a composite multifunctional coating to modulate the host response, considering the reservoir made of titanium. Iacovacci et al. focused on the second and third issues, by comparing the employment of Nylon 6 (i.e., hydrophilic) and polytetrafluoroethylene (PTFE; i.e., hydrophobic), as potential candidate polymers to fabricate insulin reservoirs by means of lathe and milling-based manufacturing systems [13]. Their work demonstrated the influence of the number of surface discontinuities on insulin aggregation, beside hydrophilic/hydrophobic properties. Among the technologies that could be used for reservoir fabrication, 3D-printing of bioinert thermoplastic polymers can address microscale surface features and be used to produce an insulin reservoir with the desired shape. Additive manufacturing approaches allow, indeed, a customized shaping of materials with fewer constraints than traditional fabrication processes [39]. Having suitable materials for insulin reservoir development would contribute to the advancement towards an AP device, which can impact millions of people around the globe.

In this study, we also focused on the second and third issues by investigating different polymers and manufacturing technologies. Indeed, we provided a complete characterization of two COC grades in view of possible employment as bulk materials for an insulin reservoir, because of their declared biocompatibility properties, bioinertness and possibility to be processed from the molten state. In particular, we analyzed the T5013 and T8007, which are characterized by a different norbornene content (i.e., 75% vs. 65%, respectively), from a thermomechanical point of view, to identify the best COC candidate for the reservoir 3D-printing. 

The analysis of the mechanical properties showed that the higher the norbornene content, the lower the ethylene sequences, and, consequently, the higher the Young’s modulus and the shorter the elongation at break [40]. This occurred for the T5013, whose stiffness was slightly higher than that of the T8007 (2.7 GPa vs. 2.5 GPa, respectively). However, from the mechanical standpoint, the T8007 was preferred over the T5013, because of its higher toughness (18 kJ/m^2^ vs. 10 kJ/m^2^) and ultimate strength (56 MPa vs. 40 MPa). The preference towards the T8007 was also corroborated by the thermal properties. In fact, the T8007 showed a lower T_g_ than the T5013 (75 °C vs. 130 °C), but it possessed significantly higher thermal conductivity, specific heat, and diffusivity than the other COC, thus leading to a lower amount of energy required to melt the filament and better quality of the 3D-printing process. The increase in norbornene content in COC is usually correlated with a linear increase in the T_g_, which was in agreement with our results [37].

In contrast, the T_os_ and T_p_ of degradation for both the COC grades were similar (~450 °C), and these properties can be attributed to their chemical and microstructure features (i.e., branching and the steric nature of the polymer chain) [41]. As the degradation temperatures (T_e_) are in the order of 480 °C, the chemical structure and microstructure of COC may play a significant role, since the branching nature of the cyclic structure increases the reaction rate but, in contrast, their chain stiffness hampers the mobility of the polymer chains, thus decreasing the reaction rate and improving thermal stability [42]. In general, the difference between the thermal and mechanical properties of the two COC grades depends on the ratio of cyclic monomer (i.e., typically, norbornene) to olefin (i.e., ethylene): the higher is the norbornene content, the stiffer is the main chain with the substitution of ethylene units by the bulky ring structure [43]. To fabricate an insulin tank to be permanently implanted in the human body, the biomaterial strength and resistance to impacts is of utmost importance, since any accidental break of the device would cause a massive release of insulin, thus a fatal hypoglycemic event.

As a result of the thermomechanical characterization of the two COC grades, we decided to fabricate a prototype of the insulin reservoir using T8007, which therefore was used to perform insulin aggregation tests. Concerning the assessment of this relevant property, we referred to the protocol described by Iacovacci et al. in [13], which included an evaluation of the insulin aggregation effect played by surface roughness of the polymers to be used for the reservoir and subsequent UV analysis to determine insulin aggregation. The first analysis served to discriminate how the fabricated smooth/rough textures can affect the hydrophobic properties and the formation of localized areas that may favor the aggregation of the insulin. This set of information was finally cross-checked with the results of the UV spectra analysis over time. In this framework, we achieved interesting results, showing that COCs may offer an alternative to Nylon 6 and PTFE for insulin storage tanks. It is well known that insulin can aggregate by forming fibrils due to environmental conditions, such as mechanical stirring, temperature, pH and concentration, which can be controlled to a certain extent in an implanted reservoir [44]. It is also demonstrated that biomaterial-insulin interaction can lead to the formation of fibrils, due to several factors, including surface roughness and hydrophobicity, which are invoked in air bubble trapping at the surface in touch with fibrin [21,23]. Clotting is harmful as can hinder the proper delivery of the therapeutic molecule and make the glycemic control inefficient. Therefore, controlling the reservoir topography can be vital to ensure a long-term function of an AP. Differently from Nylon 6, COCs are hydrophobic in nature [45], thus worse insulin aggregation outcomes could be expected. The quality of the surfaces obtained using the 3D-printing process displayed a roughness (R_a_) similar to that reported for Nylon 6 and PTFE, even though with profiles varying in a wider range (−20/+20 µm vs. −10/+10 µm, from the average value) than those described by Iacovacci et al. [13].

Differently from the abovementioned protocol, in our study insulin aggregation tests were performed mainly in static conditions to decouple the mechanical stirring from the chemical and topographic factors. In fact, the authors performed the experiment using a continuous stirring of the tank at 37 °C, which aimed to mimic harsh conditions for insulin aggregation, but it inherently included different factors imputed in this phenomenon, i.e., chemistry, topography, and mechanical agitation [13]. Although our environmental conditions could have favored the aggregation of insulin due to the rough and hydrophobic nature of the COC device surface, we demonstrated that the absorbance at 350 nm (A350), corresponding to fibril-induced turbidity, was not different from that of the insulin in its commercial vial until 14 days. The values obtained were lower values than those of Nylon 6 and PTFE. Specifically, using T8007 we achieved a value of A350 equal to 0.12 on day 14 while, in contrast, PTFE and Nylon 6 devices reached similar numerical values already on day 5. This difference could be induced by the mechanical stirring applied in that study; therefore, further investigation would be necessary to better compare T8007 COC to Nylon 6 [13]. Moreover, to further validate our approach, we carried out tests using a thermostatic bath at 37 °C endowed and oscillating plate. Additionally, in this case, the absorbance measured always showed values close to zero, meaning that no agglomeration occurred.

Our result, along with the outcomes obtained from the surface pattern analysis, is particularly interesting, since we demonstrated that local irregularities of surface had less impact on insulin aggregation than a global evaluation of the pattern (i.e., roughness). In this view, additive manufacturing technologies, such as 3D-printing, could offer intriguing options for insulin reservoir fabrication.

Finally, the main limitation of this study concerns the geometry of the reservoir. In fact, the final geometry will be different since it will have to comply with the overall topological constraints of the artificial pancreas apparatus. Therefore, we expect that the designers will have to provide a particular attention to any topological feature that might favor the agglomeration of insulin.

## 5. Conclusions

In this study, we provided a detailed characterization of two COC grades for their employment as bulk material for an implantable insulin reservoir. Between the two, Topas T8007 was identified as the best material to fabricate a biocompatible device to store insulin, because of its physico-chemical and mechanical properties (i.e., superior mechanical strength and impact resistance), which enabled an optimal manufacturing of the reservoir and superior functional performance. The 3D-printed T8007 showed excellent ability to prevent insulin aggregation up to 14 days in static conditions, which was comparable to that observed in the commercially stored insulin vials. In these experimental conditions, the manufactured T8007 (i.e., an inherently hydrophobic polymer) showed that local irregularities of the surface had less impact on insulin aggregation than a global evaluation of the pattern (i.e., roughness). These findings may disclose new opportunities in developing insulin reservoirs for AP, in terms of biomaterials and manufacturing technologies.

## Figures and Tables

**Table 1:** Processing parameters for the two COC grades.

Material	Extrusion Temperature (°C)	Screw Speed (rpm)	Cycle Time (s)	Injection Temperature (°C)	Injection Pressure (bar)	Molding Time (s)	Mold Temperature (°C)
T5013	250	100	60	250	700	20	140
T8007	230	100	60	230	650	20	80

## Data Availability

Data are available upon request to the Corresponding Authors.
